# Enhancement of Lipid Extraction from Soya Bean by Addition of Dimethyl Ether as Entrainer into Supercritical Carbon Dioxide

**DOI:** 10.3390/foods10061223

**Published:** 2021-05-28

**Authors:** Hideki Kanda, Yuji Fukuta, Wahyudiono, Motonobu Goto

**Affiliations:** Department of Materials Process Engineering, Nagoya University, Furo-cho, Chikusa, Nagoya 464-8603, Japan; fukuta.yuji.ss@gmail.com (Y.F.); wahyudiono@b.mbox.nagoya-u.ac.jp (W.); goto.motonobu@material.nagoya-u.ac.jp (M.G.)

**Keywords:** supercritical fluid extraction, subcritical fluid extraction, triglyceride, co-solvent

## Abstract

Soya beans contain a variety of lipids, and it is important to selectively separate neutral lipids from other lipids. Supercritical carbon dioxide extraction has been used as an alternative to the selective separation of neutral lipids from soya beans, usually using non-polar hexane. However, supercritical carbon dioxide extraction has a high operating pressure of over 40 MPa. On the other hand, liquefied dimethyl ether extraction, which has attracted attention in recent years, requires an operating pressure of only 0.5 MPa, but there is concern about the possibility of an explosion during operation because it is a flammable liquefied gas. Therefore, this study aims to reduce the operating pressure by using a non-flammable solvent, supercritical carbon dioxide extraction mixed with liquefied dimethyl ether as an entrainer. The extraction rate and the amount of neutral lipids extracted increased with increasing amounts of added liquefied dimethyl ether. In the mixed solvent, the amount of neutral lipids extracted was higher at an operating pressure of 20 MPa than in pure supercritical carbon dioxide extraction at 40 MPa. The mixing of liquefied dimethyl ether with supercritical carbon dioxide allowed an improvement in the extraction of neutral lipids while remaining non-flammable.

## 1. Introduction

Supercritical fluid extraction is a separation method that uses supercritical fluid as the extraction solvent. Supercritical CO_2_ (SC-CO_2_) is the most commonly used extraction solvent for supercritical fluid extraction and is neither toxic nor flammable. It exhibits selectivity because of its low viscosity, high diffusivity, and density between that of a liquid and a gas [1,2,3,4]. SC-CO_2_ is suitable for the extraction of non-polar substances; for example, vegetable neutral lipids such as C16:0, C18:0, C18:1, and C18:2 have been selectively extracted from *Vitellaria paradoxa* Gaertn seeds [2]. These studies have shown that the higher the operating pressure of SC-CO_2_, the higher the extracted amounts, and such studies have been carried out at a maximum pressure of 40 MPa, although this is dependent on the mechanical strength of the equipment required for experiments. However, the high extraction pressure increases the cost of the equipment and prevents the method from being easily implemented.

One way to increase the amount of SC-CO_2_ extracted is to mix a liquid solvent with SC-CO_2_ as an entrainer. The entrainer can be ethanol, water, benzene, hexane, ethyl acetate, or acetone. However, the advantage of the high selectivity of SC-CO_2_ may be lost. Moreover, the use of an entrainer creates the following problems: the entrainer remains in the extract or residue, the air in the working environment is contaminated if the entrainer is toxic, and improvement of the extraction capacity is limited if the solubility of the entrainer in SC-CO_2_ is low, as is the case with water. For example, when extracting polar substances such as phenolic compounds, polar solvents (methanol or ethanol) are mixed with SC-CO_2_ as an entrainer [3,4]. Methanol is toxic, and the use of ethanol may be undesirable to some customers based on religious grounds. Some of these entrainers cannot be used for food processing or need to be completely removed from the extract [3,4]. In addition, conventional research related to entrainers is mostly aimed at extracting polar substances that cannot be extracted with pure SC-CO_2_, not at increasing the yield or reducing the operating pressure of non-polar substances that SC-CO_2_ can inherently extract.

This raises a question about entrainers. Originally, the main advantages of SC-CO_2_ extraction were that there is no residual solvent, it is non-toxic and safe to release into the atmosphere, and the extraction is highly selective. To solve the problem of entrainers, a non-toxic substance that is gaseous at room temperature and pressure should be used as an entrainer. The reason why there are no examples of such research is that no such convenient entrainer has been reported so far.

In order to reduce the operating pressure while maintaining high selectivity and increasing the extraction yield of SC-CO_2_ extraction, we decided to use a low-cost substance with a low boiling point, similar solvent properties to SC-CO_2_, very low toxicity, and suitable for industrial use, as an entrainer. If the candidate substance is flammable, it is also important that the mixing ratio with SC-CO_2_ is sufficiently low and that the mixing solvent is non-flammable. We focused on dimethyl ether (DME) as a candidate solvent.

Note that DME is not diethyl ether, which is commonly known as ether. DME is the simplest form of ether, with characteristics that include a low normal boiling point (−24.8 °C) [5]; therefore, DME is liquefied when it is used as an extraction solvent [6,7,8,9,10]. There is no residual DME in the extracted materials at normal temperatures after extraction [8,9]. DME has also been approved as a safe extraction solvent for food production and as a food ingredient [11,12]. Bioassay evaluation of DME dissolved water by culturing microorganisms has been conducted, and it has been confirmed that there is no biological toxicity [13]. Additionally, DME has an outstanding characteristic along with CO_2_ in that it has zero ozone depletion potential, low global warming potential, and non-toxicity [14]. Therefore, DME is attracting attention as a new green solvent [15].

When lipids were extracted from microalgae and labyrinthulea using liquefied DME (0.51 MPa, 20 °C) and SC-CO_2_ (40 MPa, 60–120 °C) as solvents, the amount of extracted lipids was higher with liquefied DME than with SC-CO_2_ [8,10]. In the case of extraction of pungent compounds from chili powder and black pepper, the extraction amount per solvent weight was higher in DME (40 bar, 313 K) than in SC-CO_2_ (300 bar, 313 K) [16]. This indicates that their solubility in DME is higher than in SC-CO_2_. This is because the intermolecular interactions with DME are stronger than those with CO_2_. For example, DME is known to form weak hydrogen bonds. DME dimers have small triply hydrogen-bonded dimers with three C–H···O–C improper bonds [17]. Therefore, the critical point of DME (DME 400 K; CO_2_ 304 K) is higher than that of CO_2_, the critical pressure (DME, 5.24 MPa; CO_2_, 7.38 MPa) is lower than that of CO_2_, and the weak hydrogen bonds of DME are expected to be stronger than the intermolecular interactions of CO_2_. These characteristics suggest that the use of liquefied DME as an entrainer for SC-CO_2_ has the potential to reduce the operating pressure of SC-CO_2_ and to increase the extraction amount.

However, DME was originally developed as a synthetic fuel for use in both liquid and gaseous forms. In China, DME is synthesized using small-scale coalfields of low commercial value and produced at a cost equivalent to that of imported liquefied petroleum gas [18]. The price is a reasonable fuel equivalent, but the explosion range of pure DME in the air is 3.427 vol% [19]. Therefore, when DME is used as an extraction solvent, a major problem arises that it is a flammable gas. For example, leakage of DME cannot be completely prevented during the filling and removal of target materials from the extraction vessel, and explosion protection is therefore necessary. This increases the cost of the equipment. Although DME is a combustible gas, DME can be mixed with SC-CO_2_ at any ratio [20]. For example, although not under supercritical conditions, at low temperatures of approximately 100 K, liquefied dimethyl ether and liquefied carbon dioxide form a 1:1 complex. The complex is formed through the interaction of the dimethyl ether oxygen atom with the CO_2_ carbon atom, with the CO_2_ perpendicular to the 2-fold axis of dimethyl ether, in the plane of the heavy atoms of the latter [21]. The explosion range can be narrowed by mixing it with CO_2_. When the mole fraction of CO_2_ is 0.882 or more, it is no longer in the explosive range and becomes non-flammable [19]. This means that the flammable character of liquefied DME is not a problem if the amount added to SC-CO_2_ is appropriate. Already, the mixture of liquefied DME and high-pressure CO_2_ is used industrially as a foaming agent in making polystyrene foam [22], and the use of DME mixed with CO_2_ is an effective approach in practical applications. In addition to the flammability issue, SC-CO_2_ and hexane are highly selective in extracting non-polar substances because they are non-polar, while DME is weakly polar and is known to extract also polar substances when the sample is wet [23].

In this attempt to use DME as an entrainer for SC-CO_2_ extraction, we focused on the soya bean, a plant with many research examples of SC-CO_2_ extraction. Soya bean is one of the best sources of high-quality vegetable protein and oil. The use of SC-CO_2_ extraction as an alternative to organic solvents in soya bean oil extraction has been reported by several authors [24,25,26,27,28,29,30,31]. In the most recent studies, the extraction of lipids from soya beans with SC-CO_2_ [31] at 300 bar and 50 °C achieved a 6.59% yield. There is only one previous study on the extraction of lipids from soya bean “scum” using liquefied DME, which gave a 0.97% yield [32], less than that of SC-CO_2_. However, when the effects of species differences and individual differences are included, it is common for results to be very different, and this comparison cannot be used to judge SC-CO_2_ as superior.

In this study, pure SC-CO_2_ and a mixture of SC-CO_2_ and liquefied DME in such proportions to make it non-flammable were tested for the extraction of lipids from soya beans. The effects of extraction time, temperature, pressure, and mixing ratio of liquefied DME on the extraction performance were investigated.

## 2. Materials and Methods

### 2.1. Samples and Chemicals

**Figure 1:**
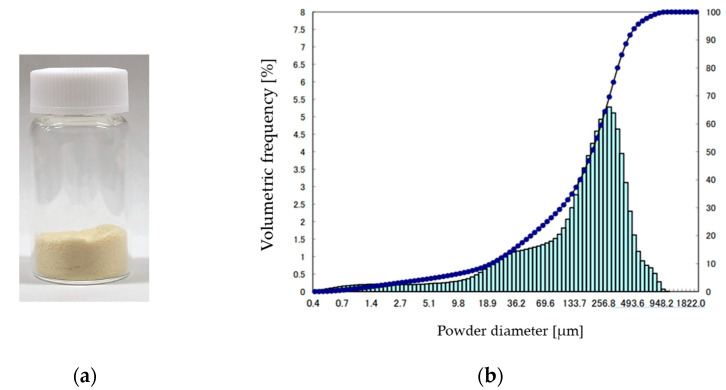
(**a**) Appearance of soya bean powder; (**b**) Particle size distribution of soya bean powder. The bar graph shows the derivative, and the line graph shows the integral.

Soya bean powder was supplied by Nishio Seifun Co., Ltd. (Nishio City, Japan). The appearance of the soya bean powder is shown in Figure 1a. The soya beans are non-GMO soya beans from the USA, and the powder is pale yellow. The particle size distribution shown in Figure 1b was measured using a particle size distribution analyzer (LA-920, HORIBA, Ltd., Kyoto, Japan). The medium diameter and most frequent diameter were 213.0 µm and 295.5 µm, respectively.

The nutrition facts disclosed by the manufacturer on the label of the soya bean powder are shown in Table 1. Carbohydrates include both sugars and fibers. Both SC-CO_2_ and liquefied DME have a poor ability to dissolve proteins and carbohydrates, and the main target for extraction is fat (lipid), which makes up 20.58% of the soya bean.

Liquefied CO_2_ was purchased from Tomoe Shokai Co., Ltd. (Tokyo, Japan) and liquefied DME (420D) from Tamiya, Inc., Shizuoka, Japan); these were used without further purification. All reagents used in thin-layer chromatography in this study were of HPLC grade (Fujifilm Wako Pure Chemicals, Osaka, Japan).

### 2.2. SC-CO_2_/DME Extraction

**Figure 2:**
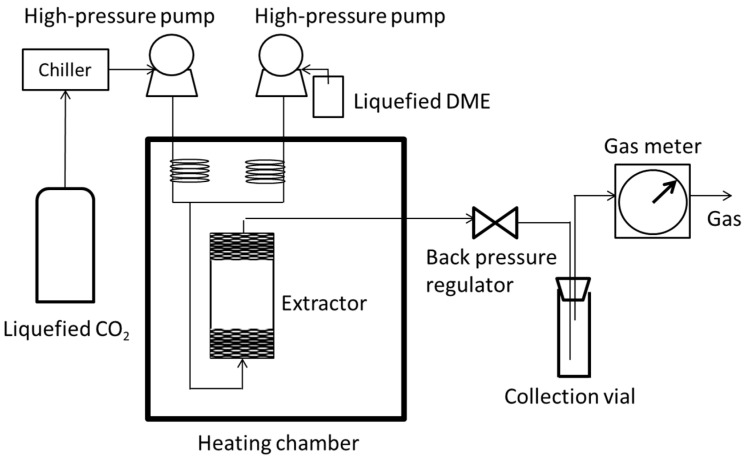
Schematic diagram of SC-CO_2_ extraction system using liquefied DME as an entrainer.

A schematic diagram of the experimental setup for the SC-CO_2_ extraction method using liquefied DME as an entrainer is shown in Figure 2.

The CO_2_ from the liquefied CO_2_ cylinder was cooled to approximately 10 °C in a chiller (TBG020AA; ADVANTEC, Tokyo, Japan) and then fed to the heating chamber using a high-pressure pump (PU-2086; Jasco, Hachioji, Japan). Similarly, a spray can of liquefied DME was fed into a heating chamber (EYELA WHO-400; Tokyo Rikakikai Co., Ltd., Tokyo, Japan) using a high-pressure syringe pump (SE260; Nikkaki-bios, Tokyo, Japan). The total flow rate of liquid CO_2_ and liquid DME was 4.0 mL/min (liquid equivalent volume). The ratios of SC-CO_2_/DME were varied at 0, 29:1, 14:1, and 9:1. Because the densities of liquefied CO_2_ at 10 °C and liquefied DME at 20 °C are 0.8611 g/mL and 0.6690 g/mL respectively, the CO_2_ molar ratios calculated on this basis are 1.00, 0.975, 0.950, and 0.924, respectively, which are not in the explosive range and the solvent mixture is non-flammable. Inside the heating chamber, the liquefied CO_2_ becomes supercritical and mixes with liquefied DME. The operating temperatures were 40, 60, and 80 °C. In the heating chamber, a SUS cylindrical extractor (17-4PH; volume 10 mL, inner diameter 2 cm; Thar Technologies, Inc., Pittsburgh, PA, USA) was filled with 3.0 g of the soya bean powder and glass beads (1.5–2.5 mm) to fix the soya bean powders above and below the extractor. The operating pressure was regulated between 10 and 40 MPa by a back-pressure regulator (BP-2080, Jasco) installed downstream of the extractor. The extraction temperature and pressure range have been examined many times in previous studies [24,25,26,27,28,29,30,31]. The mixed solvent extracted lipids from the soya bean powder in the extractor and was decompressed to gas using a back-pressure regulator. The extracted lipids were collected in a 30 mL collection vial at ambient temperature between 15 min and 4 h and analyzed immediately after extraction. The flow rate of the mixed solvent was measured using a gas meter (W-NK-1A, Shinagawa Co., Inagi City, Japan). Because there are many experimental conditions, three tests were carried out under each condition of extraction time to check the reproducibility.

### 2.3. Analysis

#### 2.3.1. Fourier Transform Infrared Spectroscopy (FTIR) Spectra

To investigate whether sufficient lipids had been extracted, FTIR spectra of the original soya powder and the extraction residue were obtained using ATR-FTIR (PerkinElmer Spectrum Two, PerkinElmer Japan K.K., Yokohama, Japan).

#### 2.3.2. Thin-Layer Chromatography (TLC)

Silica gel 60F_254_ TLC plates (layer thickness, 0.25 mm; Merck Millipore, Darmstadt, Germany) were used for crude determination of lipid classes on TLC. the extracts were dissolved in chloroform at a concentration of 5 mg/mL, placed onto the TLC plates, and developed with chloroform/methanol/water/ethyl acetate/2-propanol (5:2:1:5:5, *v*/*v*) as the mobile phase [33].

#### 2.3.3. Elemental Analysis

Elemental analysis was conducted for the extracted lipids using a CHN analyzer (CHN Corder MT-6, Yanaco Technical Science, Tokyo, Japan). The samples were burned in helium containing oxygen, NO_x_ was reduced with Cu, and the ratios of carbon, hydrogen, and nitrogen were determined from the concentrations of CO_2_, H_2_O, and N_2_ produced, while the proportion of oxygen was estimated from the difference with the total weight [34].

#### 2.3.4. Fatty Acid Analysis

During acid-catalyzed transesterification, the fatty acid-containing lipid fractions in the extracted lipids were converted to fatty acid methyl esters (FAMEs) using a fatty acid methylation kit (06482-04; Nacalai Tesque, Kyoto, Japan). Furthermore, the composition of the FAMEs was identified using gas chromatography-mass spectrometry (GC–MS; 7890A GC system and 5975C inert XL MSD with a triple-axis detector, Agilent Technologies Japan, Ltd., Hachioji, Japan) with a phenyl arylene capillary column (DB-5MS; 30 m × 250 µm (internal diameter) × 0.25 µm, Agilent Technologies Tokyo Ltd.) according to the NIST mass spectral database and quantified based on a FAME standard (Supelco 37 Component FAME Mix; Sigma-Aldrich St. Louis, MO, USA) [34]. The oven temperature was initially set at 100 °C for 5 min, increased to 270 °C at a rate of 2 °C min^−1^, and then held for 5 min. The inlet temperature was 250 °C, and the detection temperature was 300 °C. The mass range was 50–500 *m*/*z*.

## 3. Results

### 3.1. Effect of Extraction Conditions on Lipid Extraction

**Figure 3:**
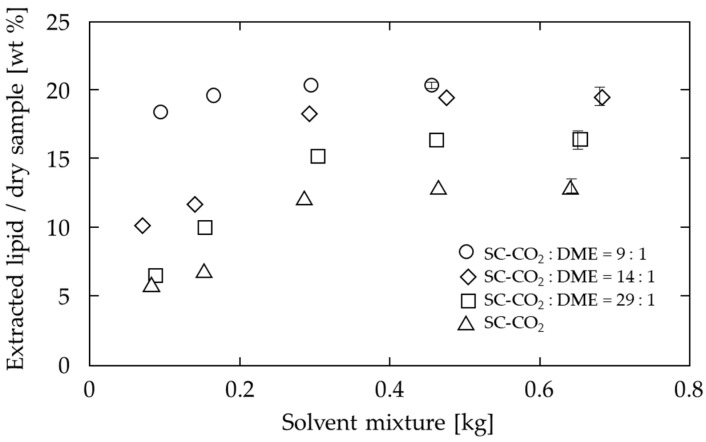
Effect of DME mixing ratio on lipid extraction from soya bean by SC-CO_2_ at 60 °C and 20 MPa.

The extraction behavior of lipids from soya bean powder at various mixing ratios of SC-CO_2_ to DME is shown in Figure 3. The temperature and pressure were 60 °C and 20 MPa, respectively, and the extraction was terminated when the extracted amount became almost constant. In the early stage of extraction, when the amount of solvent flowed was less than 0.1 kg, the amount extracted per solvent (the inclination in the figure) was very high, especially at a ratio of 9:1, the yield reached 18.5% at a solvent volume of 0.094 kg, which is 91.6% of the final total extracted amount described below. Thereafter, as the solvent flowed, the amount extracted per solvent decreased. The yields, which are extracted lipids amount based on dry sample weight, at the end of extraction were 12.9% at a ratio of 0, 16.7% at 29:1, 19.1% at 14:1, and 20.2% at 9:1. There was also a significant difference in the rate of extraction: for a solvent consumption of approximately 0.1 kg, the extraction rate was 5.85% for a ratio of 0, 6.49% for 29:1, 10.1% for 14:1, and 18.3% for 9:1, suggesting that the solubility of lipids in each solvent is different. Under all conditions, when the solvent mixture exceeded 0.4 kg, there was no change in the extraction volume, so the time required to flow above 0.4 kg (=116.1 to 118.8 min) was determined to be sufficient to complete the extraction.

**Figure 4:**
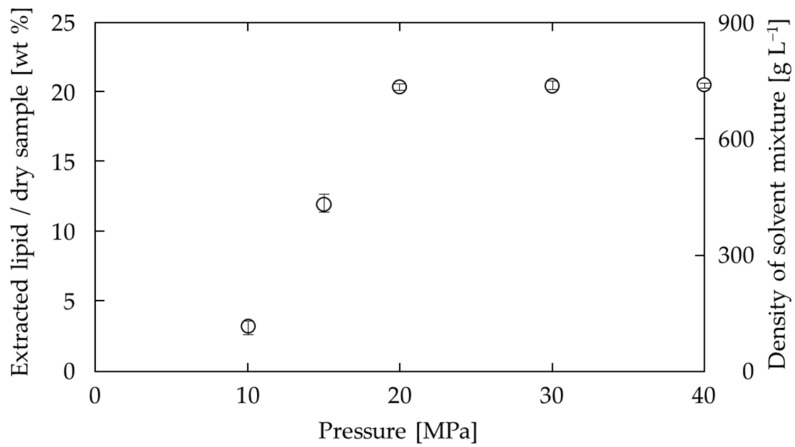
Effect of pressure on lipid extraction from soya bean by SC-CO_2_ with DME (9:1) at 60 °C. The solid curve is the density of the solvent mixture.

The effect of the pressure of the solvent mixture on the amount of extracted lipids is shown in Figure 4. At 10 MPa and 15 MPa, the amount of lipids extracted is low, but it reaches a maximum at 20 MPa, above which pressure the lipid amount does not increase. Similarly, extraction with SC-CO_2_ at 60 °C at 20, 30, and 40 MPa gave yields of 12.3%, 15.8%, and 19.1%, respectively. In other words, extraction at 20 MPa with DME at 9:1 ratio yielded more lipids than extraction with SC-CO_2_ at 40 MPa.

**Figure 5:**
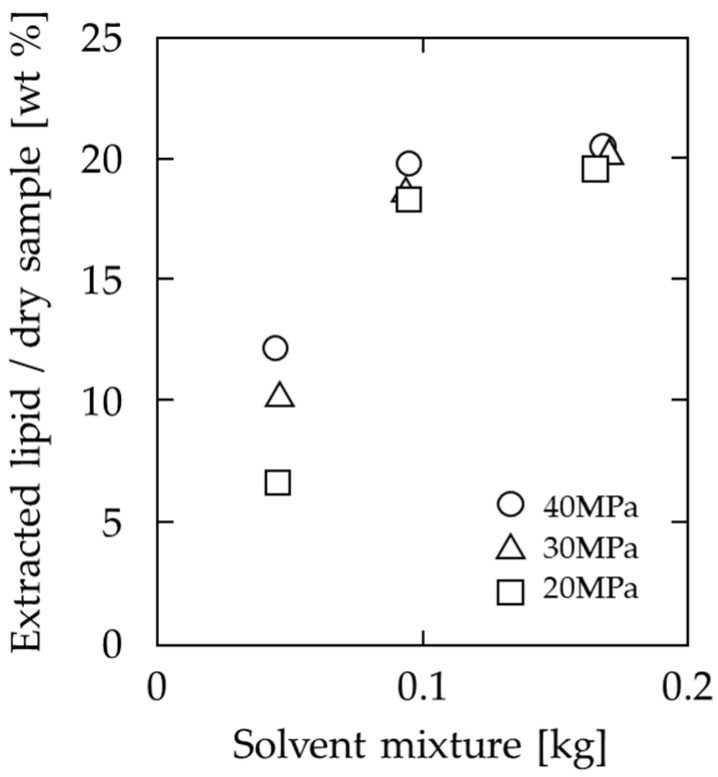
Extraction behavior of lipids from soya beans in the early stages of extraction at 20–40 MPa SC-CO_2_ with DME (9:1) at 60 °C.

Although there was no difference in the final extraction amount there was a difference in the amount extracted per solvent, and the change over time is shown in Figure 5. Similar to Figure 4, the SC-CO_2_ to DME ratio is 9:1 and the temperature is 60 °C. Figure 5 shows that at the initial stage of extraction, the higher the pressure, the higher the amount of lipids extracted. For example, when the solvent mixture flows approximately 0.044 kg, the extracted lipid amounts were 6.7 wt%, 10.4 wt%, and 12.2%, at 20, 30, and 40 MPa, respectively.

**Figure 6:**
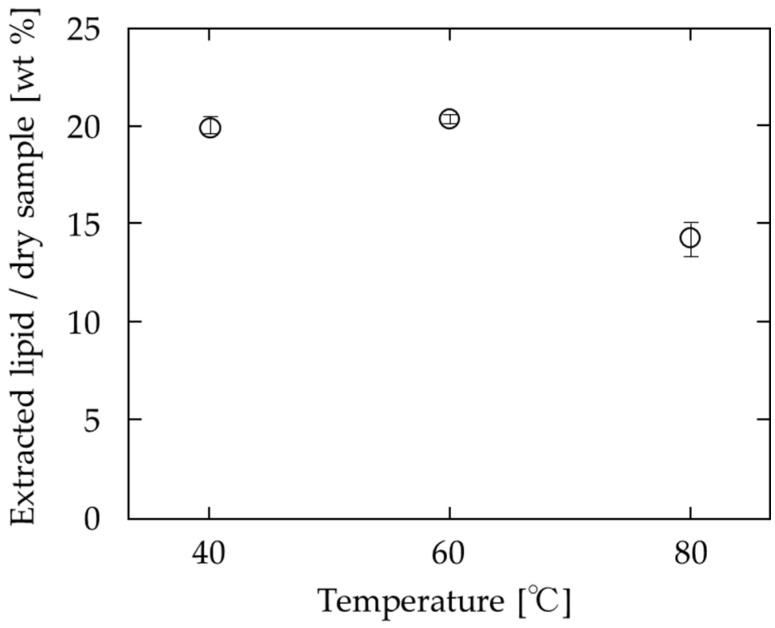
Effect of temperature on lipid extraction from soya bean by SC-CO_2_ and DME (9:1 ratio) at 20 MPa.

The effect of the extraction temperature using an SC-CO_2_/DME ratio of 9:1 and 20 MPa is shown in Figure 6. Extraction at 80 °C significantly reduced the amount of extracted lipids compared to that at 40 °C and 60 °C. This temperature dependence is because of solvent density and lipid vapor pressure on the temperature dependence. The details are described in Section 4.1.

### 3.2. Properties of the Extracts and Residue

Based on the results so far, the extraction of SC-CO_2_ with DME has shown that (1) there is no difference in the final extraction amount when the pressure exceeds 20 MPa, and (2) the temperature dependence is similar at 40 °C and 60 °C, but the extracted amount decreases at 80 °C (20 MPa), and (3) increases with an increase in the amount of DME in the mixed solvent.

As it is very difficult to analyze the products for all conditions, the results of FTIR, TLC, elemental analysis, and fatty acid analysis of the residues and lipids obtained by extraction for a sufficient time at 20 MPa, 60 °C, and an SC-CO_2_/DME ratio of 9:1, which are the conditions under which the most lipids were extracted, are presented in this section. These results were then compared with those of the original soya bean powder and the products obtained with pure SC-CO_2_ at 40 MPa and 60 °C.

**Figure 7:**
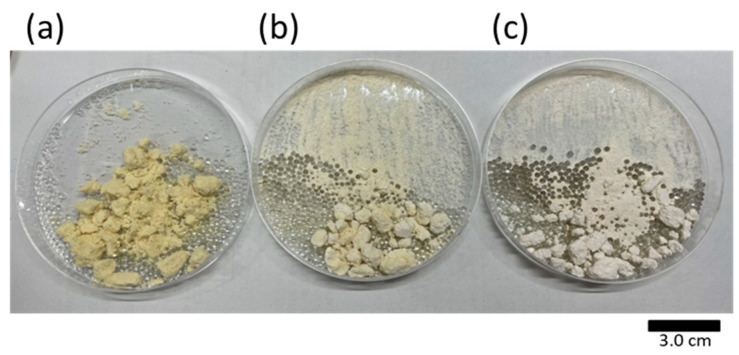
Photo images of (**a**) the original soya bean powder, and residues after extraction with (**b**) SC-CO_2_ extraction and (**c**) SC-CO_2_ with DME.

The appearance of the residues obtained is shown in Figure 7. The original soya bean powder is also shown for comparison. The yellow or white powder is the soya bean powder, and the transparent spheres are the glass beads that were added to the extractor. The powder was yellow before extraction and became lighter in color after extraction.

**Figure 8:**
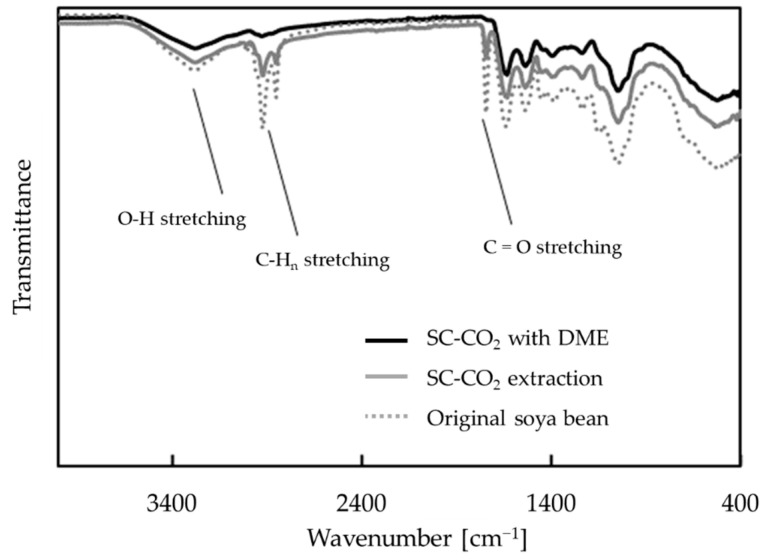
FTIR spectra of soya bean powder before extraction (gray dotted curve), and after extraction with SC-CO_2_ (gray solid curve) and SC-CO_2_ with DME (black solid curve).

The FTIR spectra of the original soya bean powder and the residues obtained are shown in Figure 8. The residue after each extraction shows little change in the fingerprint region and significant change in the functional group region. From the analysis, it is observed that the peaks around 1765–1715 cm^−1^, 2800–2950 cm^−1^, and 3400–3200 cm^−1^ decrease after SC-CO_2_ and SC-CO_2_ with DME extraction. In particular, these peaks almost disappear in SC-CO_2_ with DME.

**Figure 9:**
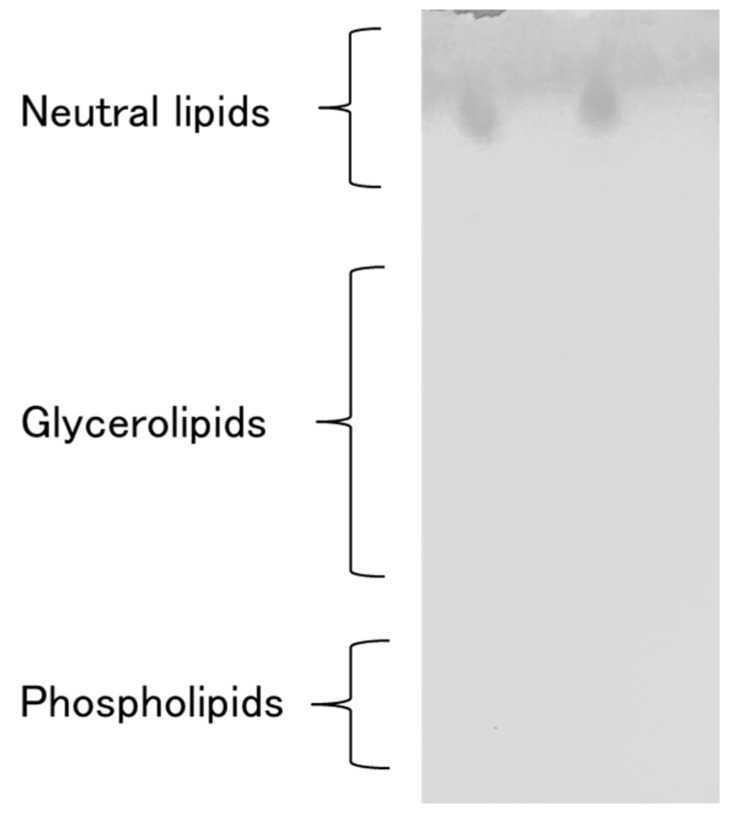
TLC images of the extracts by SC-CO_2_ (left) and SC-CO_2_ with DME (right).

As shown in Figure 9, the extracts obtained by SC-CO_2_ (left) or SC-CO_2_ with DME (right) extractions were developed on a silica gel TCL plate. Both extracts contain neutral lipids with an R_f_ value (rate of flow indicated by the distance traveled of a component compared to that of the mobile phase) of approximately 0.95–1.00.

The elemental composition of the extracts obtained by SC-CO_2_ and SC-CO_2_ with DME is listed in Table 2. Both the lipids obtained by SC-CO_2_ extraction and SC-CO_2_ with DME extraction contain 77% carbon, 12% oxygen, 10% oxygen, and almost no nitrogen.

The fatty acid composition of the extracts obtained using SC-CO_2_ and SC-CO_2_ with DME is shown in Table 3. The lipids of SC-CO_2_ and SC-CO_2_ with DME both contain mainly C16:0, C18:0, C18:1, and C18:2, and the proportions of these lipids do not change significantly. The amounts of fatty acids detected in 1 g of extracted lipid are shown in Figure 3. For all fatty acids, the extracted amounts were increased by SC-CO_2_ with DME. These results indicate that the addition of DME increases the extraction amounts while maintaining the composition of fatty acids in SC-CO_2_ extraction.

## 4. Discussion

### 4.1. Effect of Extraction Conditions on Lipid Extraction

First, as shown in Figure 3, the increase in the amount of lipids extracted from the soya bean powder with an increasing proportion of DME in the solvent mixture can be attributed to the strong intermolecular interaction of DME. This means that a ratio of 14:1 is sufficient to extract most lipids, but a ratio of 9:1 is desirable to improve solubility and reduce extraction time.

Second, the effect of the pressure of the solvent mixture on the amount of lipid extracted is related to the density of the solvent mixture. The solid curve in Figure 4 shows the pressure dependence of the density of the solvent mixture (SC-CO_2_ with DME (9:1), 60 °C). The density of the solvent mixture increases significantly around 10–20 MPa, and above 20 MPa, the increase in density is small. The pressure dependence of the density shows a similar trend to that of the pressure dependence of the amount of lipid extracted.

The amount extracted per volume of solvent at the beginning of the extraction, i.e., the slope in Figure 5, suggests that solubility is important. Above 20 MPa, the slope suggests that a higher pressure results in higher solubility of lipids in the solvent mixture. However, the final amount of lipid extracted was almost the same above 20 MPa, also suggesting that an operating pressure of 20 MPa is sufficient to obtain solvent-lipid interactions that are much stronger than soya bean powder-lipid interactions.

The temperature dependence of the extraction volume, shown in Figure 6, can be attributed to the large change in the density of SC-CO_2_ with changing temperature and pressure. In general, the solubility of a solute in a solvent is strongly influenced by the density of the solvent and the saturation vapor pressure of the solute [34]. Increasing the temperature at constant pressure increases the solubility of lipids, as they evaporate more easily. At the same time, however, the density of SC-CO_2_ decreases, which weakens the interaction between carbon dioxide molecules and lipids and hence decreases their solubility. The condition at which the temperature dependence of solubility is reversed in SC-CO_2_ is commonly known as the crossover point. In the present study, a crossover point was observed because the contribution from the decrease in the density of the solvent mixture was more significant than that from the increase in the saturated vapor pressure of lipids during the increase from 60 to 80 °C. In other words, a crossover point exists in the mixture of SC-CO_2_ and DME, as well as in pure SC-CO_2_.

Based on the results so far, the extraction of SC-CO_2_ with DME has shown that (1) there is no difference in the final extraction amount when the pressure exceeds 20 MPa, and (2) the temperature dependence is similar at 40 °C and 60 °C, but the extracted amount decreases at 80 °C (20 MPa), and (3) increases with an increase in the amount of DME in the mixture.

### 4.2. Properties of the Extracts and Residue

First, the different colors of the extracts, shown in Figure 7, are due to the yellow color of the soya lipids contained in the soya beans, which are whiter in the mixed solvent with DME than in the extraction with SC-CO_2_ alone, suggesting that more lipids were extracted in the former case.

The FTIR peaks shown in Figure 8, which are less intense in the extraction residue than in the original soya bean powder, correspond to C=O, C–H_n_, and O–H bonds [35,36], all of which are present in fatty acids [37]. The decrease in the peak heights was more pronounced with the addition of DME, suggesting an increase in the amount of lipids extracted.

In the TLC analysis shown in Figure 9, the extracts exhibited an R_f_ value of 0.95 to 1.00. In a previous paper, this value has been reported as being characteristic of neutral lipids [38]. Phospholipids (Rf ≈ 0) and glycerolipids (Rf = 0.16–0.61) were not significantly detected. In other words, the addition of DME to SC-CO_2_ did not impair the selectivity of SC-CO_2_.

The components of the extracted lipids were identified from the results of elemental analysis shown in Table 2. For comparison, the elemental composition of triglycerides (TG), which are composed of three molecules of linoleic acid (C18:2), one of the main components of lipids, is also given. The elemental compositions of monogalactosyldiacylglycerol (MGDG) and phosphatidylcholine (PC) are also given. The results of the elemental analysis show that both SC-CO_2_ and SC-CO_2_ with DME extractions are in close agreement with the elemental composition of C18:2 triglycerides. On the other hand, they are clearly different from the elemental compositions of MGDG and PC. This demonstrates that a selectivity similar to that of SC-CO_2_ is maintained when DME is added. Similarly, the fatty acid composition shown in Table 3 indicates that DME is a suitable entrainer for obtaining similar lipids without compromising the selectivity of SC-CO_2_.

These results can be explained by the fact that the Hansen solubility parameters (HPS) of CO_2_ and DME are very similar. In the HPS concept, the intermolecular interactions are classified into three components: δd (the energy from dispersion forces between molecules), δp (the energy from dipolar intermolecular forces between molecules), and δh (the energy from hydrogen bonds, π–π stacking interactions, coordination bonds, and charge transfer interactions), which are used to estimate the ease of mutual solubility. The δd, δp, and δh values for DME are 15.2 MPa^1/2^, 6.1 MPa^1/2^, and 5.7 MPa^1/2^ [39], and those for CO_2_ are 15.7 MPa^1/2^, 6.3 MPa^1/2^, and 5.7 MPa^1/2^ [40]. The similarity of the properties of the extracts by SC-CO_2_ extraction and SC-CO_2_ with DME extraction is supported by the HPS concept that solutes are readily soluble in solvents that have similar three-dimensional vectors of HPS parameters [41].

## 5. Conclusions

The maximum amount of lipid extraction from soya bean powder was reached when a mixture of SC-CO_2_ and liquefied DME was used with an SC-CO_2_/DME ratio of 14:1, and the extraction rate was fastest at a ratio of 9:1. The extraction temperature was between 40 and 60 °C. The maximum extraction was reached at a pressure of 20 MPa or higher, and the speed of extraction increased at higher pressures. More lipids were extracted in the extraction operation at 20 MPa using mixed solvents than in SC-CO_2_ at 40 MPa, and DME functioned as an entrainer to reduce the operating pressure. TLC analysis, elemental analysis, and GC–MS analysis showed that the lipids obtained were neutral lipids composed of C16 or C18 components. The same high selectivity as that of SC-CO_2_ was maintained. In other words, DME added in a ratio that does not impair the non-flammability of SC-CO_2_ is very effective as an entrainer of SC-CO_2_. These features can be explained by the hydrogen bonding of DME and similar HPS parameters for CO_2_ and DME.

## Figures and Tables

**Table 1:** The nutrition facts label of the soya bean sample.

	Moisture	Protein	Fat	Carbohydrate	Ash
% Wet basis	9.86	40.39	20.58	25.09	4.08

**Table 2:** Elemental composition of extract obtained by SC-CO_2_ and SC-CO_2_ with DME.

Dry Ash Free (wt%)	Carbon (±0.3)	Hydrogen (±0.1)	Nitrogen (±0.2)	Oxygen (±0.6)
SC-CO_2_	77.5	12.0	0.2	10.3 ^1^
SC-CO_2_ with DME	77.3	12.1	0.0	10.6 ^1^
TG (C18:2)	77.6 ^2^	11.5 ^2^		10.9 ^2^
MGDG (C18:2)	71.6 ^2^	11.0 ^2^		17.4 ^2^
PC (C18:2)	10.6 ^2^	68.3 ^2^		21.1 ^2^

^1^ By difference, so other elements are included. ^2^ Calculation from a molecular formula.

**Table 3:** Fatty acid composition of extract obtained by SC-CO_2_ and SC-CO_2_ with DME.

Fatty Acid (%)	C16:0 (±0.4)	C16:1 (±0.02)	C18:0 (±0.1)	C18:1 (±0.2)	C18:2 (±0.8)	C20:0 (±0.3)	Others (±0.2)
SC-CO_2_ ^1^	18.4	0.3	10.8	20.6	47.3	1.6	1.1
SC-CO_2_ with DME ^1^	21.0	0.4	10.0	20.0	46.6	1.3	0.8
**Fatty Acid (mg/g)**	**C16:0 (±3.6)**	**C16:1 (±0.2)**	**C18:0 (±0.6)**	**C18:1 (±1.7)**	**C18:2 (±5.5)**	**C20:0 (±1.5)**	**Others (±1.3)**
SC-CO_2_	106	1.7	62.0	118	273	9.0	6.0
SC-CO_2_ with DME	150	2.6	71.5	143	335	9.0	5.9

^1^ Total does not add up to 100 due to rounding errors.
